# Risk of Lymphohematopoietic Malignancies in Uranium Miners

**DOI:** 10.1289/ehp.115-1852699

**Published:** 2007-04

**Authors:** Matthias Möhner

**Affiliations:** Federal Institute for Occupational Safety and Health, Berlin, Germany, E-mail: moehner.matthias@baua.bund.de

Řeřicha et al. (2006) analyzed data from Czech uranium miners with respect to incidence of malignancies of the lymphohematopoietic system. Their results, however, do not correspond with those of two recent studies on German uranium miners (Kreuzer et al. 2004; Möhner et al. 2006). Řeřicha et al. (2006) used a case–cohort design, in which the subcohort was stratified by attained age and duration of employment. Stratification by age is a standard approach in case–cohort studies to optimize data ascertainment in the sub-cohort. However, stratification by duration of employment is problematic, because in occupational epidemiology it should be assumed that the duration of employment is highly correlated with cumulative exposure. Therefore, this kind of stratification contradicts the general demand for a random selection of controls with respect to exposure under study. Comparing the ratios of sampling fractions (< 12 months vs. ≥ 12 months duration of employment) between age groups results in a heterogeneous picture (Table 1).

It is not uncommon in occupational cohort studies to include only subjects with a duration of employment of at least a certain number of months into the cohort. An analysis of only those miners with an employment duration of at least 12 months would be in line not only with the standard methodology but also with earlier published results of the authors (Řeřicha et al. 1998). Hence, the authors should have at least explained their reasoning for including the remaining miners in a second set of strata. In addition, they should have presented separate results for both duration strata to validate the result of the combined analysis.

Given the above-mentioned assumption concerning the relationship between duration of employment and cumulative exposure, I calculated crude incidence rate ratios using data from Table 1 of Řeřicha et al. (2006). The age-specific odds ratios cover a wide range (0.29–7.16), and a corresponding test yields only borderline homogeneity. Consequently, completeness of matching with the cancer registry needs to be discussed.

According to the study design, the time period between last exposure and begin of follow-up can span up to 27 years; therefore, the healthy-worker survivor effect could be an important issue in this study (Řeřicha et al. 2006). In light of the discussion on the magnitude of the latency period for leukemia, more detailed results would be useful to get an impression on, for example, the effect of the year of last exposure.

## Figures and Tables

**Table 1:** Rate ratios (RRs) of sampling fractions and incidence rate ratios (IRRs) for lymphohematopoietic malignancies (95% confidence intervals), calculated from Rericha et al. (2006).

Age (years)	RR sampling fractions	IRR
19–35	0.52 (0.40–0.69)	1.06 (0.47–2.61)
36–45	1.11 (0.91–1.37)	0.56 (0.27–1.19)
46–55	1.16 (1.00–1.34)	1.08 (0.63–1.95)
56–65 (1.18–292.75)	1.54 (1.21–2.00)	7.16
66–90	1.24 (0.77–2.07)	0.29 (0.05–1.99)
M-H combined	1.09 (0.99–1.20)	1.00 (0.71–1.41)
Homogeneity test	*p* = 0.000	*p* = 0.055
